# Nanostructured Porous Silicon for Bone Tissue Engineering: Kinetics of Particle Degradation and Si-Controlled Release

**DOI:** 10.3390/jfb14100493

**Published:** 2023-09-30

**Authors:** Naveen Fatima, Hamideh Salehi, Eduardo J. Cueto-Díaz, Alban Desoutter, Frédéric Cuisinier, Frédérique Cunin, Pierre-Yves Collart-Dutilleul

**Affiliations:** 1 LBN, University of Montpellier, 34000 Montpellier, France; naveen.fatima@umontpellier.fr (N.F.); alban.desoutter@umontpellier.fr (A.D.); frederic.cuisinier@umontpellier.fr (F.C.); 2 Institut Charles Gerhardt UMR 5253, CNRS-ENSCM-University of Montpellier, 34000 Montpellier, France; ecueto@cab.inta-csic.es (E.J.C.-D.);; 3 Centro de Astrobiología (CSIC-INTA), Torrejón de Ardoz, 28850 Madrid, Spain; 4 Faculty of Dentistry, University of Montpellier, 34000 Montpellier, France; 5 Service Odontologie, Centre Hospitalier Universitaire de Montpellier, 34000 Montpellier, France

**Keywords:** bone tissue engineering, bioresorbable scaffold, surface functionalization

## Abstract

Nanostructured porous silicon (pSi) is a synthetic silicon-based material. Its biocompatibility and bioresorbability in body fluids make pSi an appealing biomaterial for tissue engineering, with surfaces characteristics facilitating human cell adhesion and differentiation. The resorption kinetics of such porous biomaterials is crucial for in vivo bone regeneration, in order to adapt biomaterial resorption to tissue formation, and to control the release of loaded bioactive molecules. We investigated pSi as a bioactive scaffold for bone tissue engineering, with an emphasis on kinetics of pSi resorption and silicon release. PSi particles and chips were fabricated from crystalline silicon, and functionalized by oxidation and chemical grafting of amine groups to mimic biological structures. Materials resorption over time was investigated with Raman spectroscopy, infrared spectroscopy, and Scanning Electron Microscopy. Silicon release was followed by mass spectrometry. Particle degradation and inclusion in newly formed bone were studied in vivo. The in vitro experiments revealed that non-oxidized pSi had an accelerated initial dissolution in ddH_2_O and an inhibition of initial Si release in SBF. This high reactivity also led to transformation towards amorphous non-resorbable silica when incubated in SBF. PSi resorption started immediately with a maximal dissolution in the first 24 h. Later, the dissolution rate decreased over time. In comparison, the resorption process of oxidized pSi seemed delayed, but more continuous. This delayed dissolution increased the bioactivity and stability, leading to enhanced bone formation in vivo. Delayed pSi degradation provided a constant surge of silicic acid over time and promoted bone regeneration, demonstrating the high potential of pSi for bone tissue engineering: Oxidized pSi were almost completely resorbed after 2 months of healing, with remaining partially dissolved particles surrounded by newly formed bone. On the contrary, non-oxidized particles were still obviously present after 2 months with limited bone regeneration. This delayed resorption is consistent with the in vitro observations in SBF, and particles’ transformation towards silica.

## 1. Introduction

The nanostructured porous materials that are formed by removing material from the bulk volume frequently have properties that differ from their parent material. The modified properties are because of quantum-dimensional effects in nanomaterials and surface effects related to the appearance of new ions (electrons or protons) state on the surface. There is also a noticeable increase in surface area after pore formation. The local fields in the nanocomposite medium that are characterized by the size, shape, and order of a nanomaterial contribute to these nascent properties [1].

Nanocrystalline silicon is a form of porous silicon (pSi) with a crystalline structure, in contrast to amorphous silicon, which is the non-crystalline form of silicon. The silicon nanocrystallites are covered by amorphous silicon, which oxidizes over time upon exposure to air oxygen [2]. Porous silicon (pSi) has many applications in the fields of photonics, with a photoluminescent behavior, and for biomedical applications, due to its biocompatibility and bioresorbability [3]. These properties of biocompatibility and bioresorbability come from its ability to oxidize in aqueous solution, allowing the formation of silicon oxide. PSi has a sponge-like structure, with a monocrystalline silicon presenting a diamond face-centered lattice. The layer of silicon oxide is easily (and naturally) dissolved by body fluids [4]. And the dissolution products are non-toxically eliminated as silicic acid in the urine [5]. These steps of oxidation and dissolution can be represented as follows:

Si + O_2_ → SiO_2_ (silicon oxide)

SiO_2_ + 2 H_2_O → [Si(OH)_4_] (silicic acid)

Thus, pSi has been considered as an implantable biomaterial, for both tissue engineering (mainly bone tissue) and drug delivery device (in ophthalmology and anticancerous therapy) [6,7,8,9,10,11]. It can be used directly after synthesis from Silicon wafers and chemical surface functionalization, or as a composite biomaterial [12,13]. Besides, a major advantage of pSi, in the field of biomaterials, is its intrinsic ability to be easily degraded in aqueous solutions into non-toxic silicic acid. This resorption justifies that pSi is considered as a bioactive material, since its release of Si(OH)_4_ stimulates mineralized matrix formation [14].

The surface of pSi pore wall continuously evolves from the instant it is removed from the electrolyte and exposed to air [15]. Understanding detailed structural characterization is a key factor to enhance the knowledge of pSi properties and its transition to other subtypes, and, potentially, increase its areas of application. Several techniques can be used to characterize porous silicon and study its intricate morphology and structure [16,17]. These methods are helpful in analyzing the changes occurring during its degradation. Transmission electron microscopy (TEM), scanning electron microscopy (SEM), atomic force microscopy (AFM), energy dispersive X-ray spectroscopy (EDX), X-ray photoelectron spectroscopy (XPS), interferometric reflectance spectrometry (IRS), Raman spectroscopy, and Fourier Transform Infrared (FTIR) spectroscopy are notable among the most studied.

Freshly prepared pSi is highly unstable in ambient atmosphere and its passivated layer with hydrogen termination (–Si–H) degrades and oxidizes rapidly [18]. The resulting porous nanostructure has completely different properties to pSi. Hence, pSi’s surface should be stabilized to retain its properties. Two strategies have been developed, resulting in the formation of either silicon–oxygen or silicon–carbon bonds [19,20]. Silicon–oxygen bonds are generated by thermal oxidation in air, ozone oxidation, and oxidation by organic molecules. The silanol groups on oxidized pSi surface may be functionalized by amines. Even then the oxidized surface is prone to degradation as silicon–oxygen bonds are susceptible to hydrolysis [21]. For in vivo applications, involving pSi either as tissue engineering scaffold or drug delivery, the kinetics of dissolution in aqueous body fluids are very important.

The objective of this research project was to follow the resorption kinetics of pSi particles, in the context of bone tissue engineering, in order to determine the consistency of pSi particle use, and the potential for bioactive factors release. The experimental work focused on the degradation of thermally oxidized (with or without chemical functionalization) and non-oxidized pSi over time, in simulated body fluid and in ultra-pure water (ddH_2_O) as control. The release of silicic acid was also followed from these samples, to objectivate the input of pro-osteogenic Si and the potency of pSi particles to release entrapped biomolecules during their degradation. The degradation kinetics and silicic acid release was studied by optical microscopy, SEM, EDX, Raman spectroscopy, FTIR, and induction-coupled plasma mass spectroscopy (ICP-MS).

## 2. Materials and Methods

### 2.1. Porous Silicon Particle Production

**Figure 1:**
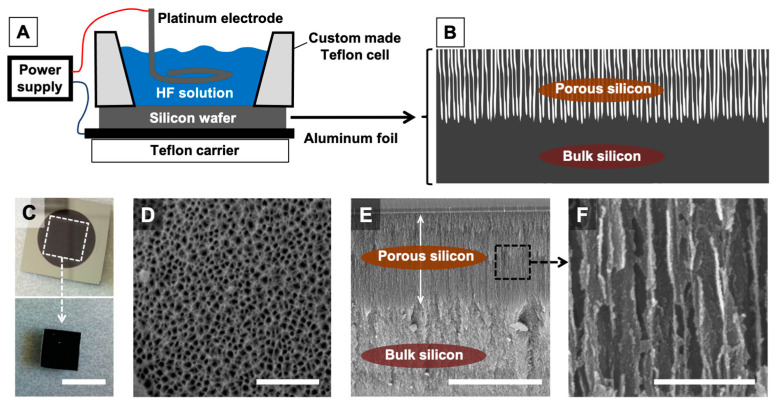
Schematic representation of the electrochemical set-up used to make porous silicon. (**A**) Schematic side view of the custom-made set-up, with chamber containing hydrofluoric acid solution (HF solution); (**B**) schematic side view of the silicon wafer after etching, with a superficial porous part (porous silicon) and an unetched part (bulk silicon). (**C**) Top view photograph of a freshly etched silicon wafer. The black area is the porous part while the mirror area is the unetched part. Scale bar = 1 cm. (**D**) SEM image of pSi surface. Scale bar = 500 nm. (**E**,**F**) SEM images of pSi: cross section of the wafer. (**E**) Scale bar = 10 μm. (**F**) Scale bar = 300 nm.

Porous silicon (pSi) wafer/chips were prepared via electrochemical etching of silicon wafers (p++ type boron-doped crystalline, 0.0012 Ωcm resistivity) in a custom-made Teflon cell. First, etching was performed at a constant current density of 200 mA/cm^2^ for 25–30 min from generator source, in a hydrofluoric acid (HF) solution in ethanol (3:1 HF: ethanol solution, *v*/*v*) which formed a porous layer on the surface. Some samples were preserved as pSi wafers and preserved in alcohol; rest were subjected to electro-polishing to yield microparticles. In order to produce pSi microparticles, second etching followed with 3.1% HF in ethanol at a current density 4 mA/cm^2^ for 4 min 10 s. The second etching leads to the detachment of formed porous layer from the Si wafer. This layer was rinsed with ethanol and collected in a glass bottle with absolute alcohol. Particles were subjected to sonication for 5 min at 25 Hz in ultrasonicator. Some of the particles were preserved in alcohol and the rest were oxidized at 400 °C for 60 min along with some pSi wafers and later submitted to silanization with aminopropyltriethoxysilane (APTES) using 200 µL APTES in 10 mL of dry toluene for 60 min. Finally, we obtained non-oxidized porous silicon wafer and particles (non-Ox pSi), oxidized porous silicon wafer and particles (Ox pSi), oxidized porous silicon wafer, and particles with silanized surface (Ox pSi with APTES). Non-etched bulk silicon (bulk-Si) was used as a control. A schematic representation of the experimental set-up and the etching process is given below in Figure 1.

### 2.2. Optical Microscopy

The degradation of all the samples were followed in phosphate-buffered saline (PBS) used as simulated body fluid (SBF) and in ultra-pure water (ddH_2_O). Samples were air-dried and analyzed after fixed intervals of time: in air, t0, t4 h, t7 h, t24 h, t48 h, t72 h, t1 week, and t2 weeks. Experiment was run in triplet.

PSi particles were incubated in PBS (pH 7.4) and in ddH_2_O (pH 6.6–7.6) for 4 weeks. Brightfield microscopy at low magnification (5×) was used to follow particle changes during the experiments. In particular, the changes in color and transparency were considered.

### 2.3. Scanning Electron Microscopy (SEM) and Energy-Dispersive X-rays Spectroscopy (EDX)

The topography of surface-modified pSi samples was analyzed via scanning electron microscopy (Analytic FEI Quanta FEG 200) to determine pore size, with an acceleration voltage of 20 kV, in a pressure of 0.5 Torr. Pore diameter was expressed for each studied area as mean diameter ± standard deviation of the mean.

Samples were observed under a FEI Quanta 200 FEG scanning electron microscope, coupled with an energy dispersive X-ray (EDX) analysis detector. The samples were placed on an adhesive carbon tape, fixed to a copper plate, and observed at an accelerating voltage of 15 kV. EDX microanalysis was employed to detect the presence of oxygen and silicon on the samples, with EDX spectra collected at 2500× magnification.

### 2.4. Induced Coupled Plasma—Mass Spectrometry (ICP-MS)

Particles were incubated in PBS or in ddH_2_O over a period of 5 weeks. The supernatant was changed and collected every second day. The Si content in all the supernatants was measured by means of inductively coupled plasma mass spectrometry (ICP-MS). ICP-MS analysis was performed by introducing extract aliquots into a mass spectrometer (Element XR ICP-MS, Thermo Scientific™, Waltham, MS, USA). Solutions were sprayed through a high-solid-type nebulizer as plasma into a thermoelectrically controlled spray chamber. The results of the analysis were then compared with a standard for identification and quantification of Si concentration. The average and standard deviation of the Si concentrations per set of samples were reported as absolute concentrations, expressed in ppm. The Si concentrations were then calculated as mg/mL. Experiments were conducted in triplicate and statistical analyses were conducted by comparing groups two by two with Student *t*-test.

### 2.5. Raman Spectroscopy

Raman spectra were collected using a Witec Confocal Raman Microscope System alpha 300R (Witec Inc., Ulm, Germany). Excitation in confocal Raman microscopy is generated by a frequency doubled Nd:YAG laser (Newport, Evry, France) at a wavelength of 532 nm. The incident laser beam is focused onto the sample through a 60× Nikon water immersion objective with a numerical aperture of 1.0 and a working distance of 2.8 mm (Nikon, Tokyo, Japan). The immersion objective decreases the manipulation time and avoids the thermal damages (degradation/degeneration) induced by high-power laser exposure. The acquisition time of a single spectrum was set to 5 s. Data acquisition was performed using Image Plus 2.08 software from Witec.

Data acquisition was conducted with Witec Control 4.0 software. At each pixel a complete Raman spectrum was recorded. The integration time per pixel varied from 0.5 to 2 s, i.e., the acquisition time per image vary from 80 min to 330 min depending on image size. Raman images were generated during data acquisition by integrating over the CH-stretching band (2800–3030 cm^−1^).

### 2.6. Attenuated Total Reflectance-Fourier Transform Infrared Spectroscopy (ATR-FTIR)

FTIR evaluation of the samples was conducted using Fourier transform infrared spectrometer Horiba Jobin Yvon—LabRAM Aramis to detect different vibrations of the oxidized transition species during degradation. Surface analysis was conducted over a spectral range of 600–4000 cm^−1^ in ATR mode—attenuated total reflectance, at a resolution of 8 cm^−1^ using the detector MCT (liquid nitrogen) and diamond objective.

### 2.7. In Vivo Experiments

The in vivo experiments were conducted on male Wistar rats, after approval by the ethical committee for animal welfare of Montpellier University (referral number 1083 16 June 2014). We used caudal vertebrae critical size defect model to investigate the effect of porous silicon on bone regeneration as previously described [22]. Animals were anesthetized with an intraperitoneal injection of ketamine and xylazine (40 and 9 mg/kg, respectively). The tail was disinfected and, after a dorsal incision, the skin and periosteum were gently retracted and the vertebrae were exposed. Intraosseous defect of 2 × 3 mm was created in the exposed surface of each of the 4 vertebrae, using a surgical guide for optimal positioning of the dental bur. The 4 bone defects were filled with respective biomaterial during different studies conducted on pSi. For in vivo experiments, we used particles of oxidized and silanized pSi, as silanization has been previously shown to promote cell adhesion [7,23], and compared them to non-oxidized pSi particles and to non-porous silicon particles (bulk silicon). After material implantation, the skin was repositioned over the defects and sutured together with a resorbable suture. Experiments were conducted in triplicate (3 rats, each rat being its control) and were sacrificed after 60 days. Rats were euthanized, and collected tails were fixed in 4% PFA for 2 days at room temperature, then kept in PBS at 4 °C until micro-Computerized Tomography (µCT) observation and histological processes.

For µCT analysis, we used a Skyscan 1172 X-ray Microtomograph (Microphotonics Inc., Allentown, PA, USA) and 3D reconstruction software (Avizo, FEI company, Hillsboro, OR, USA). Rat tails were scanned at 360° rotation at 0.7 degree intervals. Measurements were made on the Region of Interest (ROI) × 1.5 mm Tissue Volume (TV) on the computer-reconstructed 3D samples. The results were analyzed with ImageJ software for images and bone density calculations.

In order to observe mineralization and newly formed bone, non-decalcified bone samples were assessed histologically: after µCT acquisition, vertebrae were isolated using a cutting blade, then dehydrated through a graded series of ethanol treatments. Non-decalcified bone specimens were infiltrated and embedded in Technovit 9100 resin (Kulzer, Germany). Vertebrae were then cut using a precision saw with diamond disc (Isomet 4000, Buehler, Germany). Slices were polished with diamond disc and polishing cloth with diamond paste. Sections obtained were verified for the size and semi-thin sections of 100 µm were obtained. The sections were then attached to glass slides using technovit 7200 and deplasticized. The slides were colored with Masson-Goldner trichrome staining, following the sequence of immersion: hematoxylin for 10 min, rinsing with tap water, Orange G—phosphomolybdic acid for 7 min, rinsing with 1% acetic acid, light green for 40 min, rinsing with 1% acetic acid. This staining permitted us to evaluvate collagen structures (colored in green).

## 3. Results

### 3.1. Particle Dissolution: Optical Microscopy and Si Release

The etching process led to the production of pSi chips that were further detached and sonicated, in order to produce pSi particles. The experimental set-up, with views of the obtained etched chips, is presented in Figure 1.

**Figure 2:**
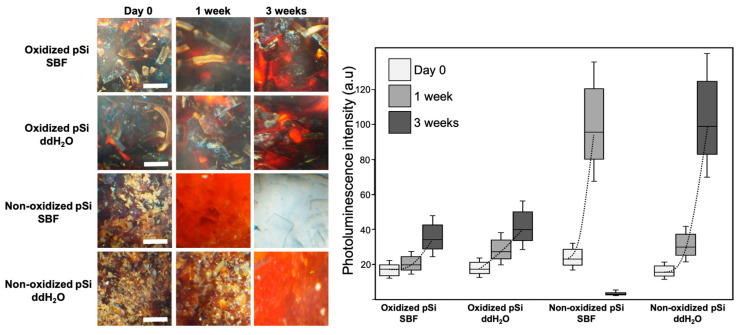
Optical microscopy and particle photoluminescence, elucidating modification over time. Scale bar = 100 µm. The histogram represents the intensity of photoluminescence of each sample initially, after 1 week, and after 3 weeks. The dash line between plots is to guide the eye, and to highlight the evolution.

The obtained particles were incubated in SBF and in ddH_2_O for a period of 4 weeks. Pictures of the samples were taken under brightfield optical microscope twice a week. Dissolution media (PBS or ddH_2_O) were changed and collected hourly until 8 h and later, every second day, and Si content was analyzed via ICP-MS. The particle color changed during the experiment, starting from dark brown for oxidized pSi and brown-red for non-oxidized pSi. The particles’ color became lighter during the experiment, towards a red color. We quantified red color intensity with ImageJ software, to follow the color changes. Oxidized particles became slightly brighter over time. Non-oxidized samples turned more rapidly towards red, with non-oxidized pSi in PBS suddenly becoming transparent after 2 weeks. Transparency was visible only for non-oxidized pSi in PBS. These results are presented in Figure 2, with optical pictures and red color quantification. During the first hours, a constant gas release was observed under binocular loupe, with a slow but continuous flow of air bubbles when non-oxidized pSi particles were immersed in PBS. This phenomenon was visible only for non-oxidized pSi particles in PBS. We also noticed that the whole batch of non-oxidized particles swelled during the first days (only in PBS with an obvious increase of volume, easily visible in a 96-well plate).

**Figure 3:**
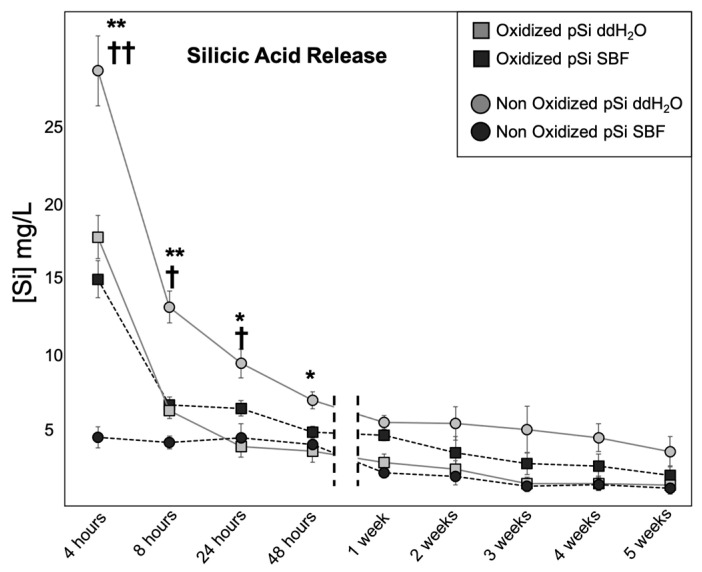
Silicic Acid release: measurements of Si in supernatant by ICP-MS. At each time point, the amount of Si corresponds to the amount released since the last measurement. (**) and (*) indicate a significant difference between non-ox pSi in ddH_2_O and non-ox pSi in SBF (*p* < 0.01 and *p* < 0.05, respectively). (††) and (†) indicate a significant difference between non-x pSi in ddH_2_O and ox pSi in ddH_2_O (*p* < 0.01 and *p* < 0.05, respectively).

When changing the dissolution media, supernatants were collected for ICP-MS analysis of Si content. The results of Si release over time are presented in Figure 3. Si release was at a maximum during the first 4 h for Ox pSi in SBF or ddH_2_O and non-Ox pSi in ddH_2_O. The amount of Si released drastically decreased after 8 h and became almost constant after 1 week, and until 5 weeks. Even though the release slightly decreased over time (from 1 to 5 weeks), we could still measure Si in the supernatant after 5 weeks. The pattern was different for non-ox pSi in SBF where the release of silicic acid was not different between 4 h, 8 h, 24 h, and 48 h. We also noticed a significant difference of release between non-oxidized particles in SBF or in ddH_2_O: the difference was obvious during the 8 first hours, and particles in ddH_2_O kept releasing more Si over time (even after 5 weeks).

### 3.2. Raman Spectroscopy

PSi chips were studied via Raman spectroscopy and ATR-FTIR over 2 weeks, when placed either in PBS or in ddH_2_O. Chips were used in these experiments to enhance the spectral signals with plane surfaces (which were difficult to obtain with particles).

All the samples were analyzed via Raman, and stacking all the spectra together showed characteristic peaks of Si–Si and Si–O bonds. The spectra presented specific features: a peak at 320 cm^−1^ (Si–O scissoring), a broad shoulder from 450 cm^−1^ to 500 cm^−1^ (corresponding to the broad 480 cm^−1^ peak of Si–Si vibration in its amorphous phase), a high-intensity peak around 520 cm^−1^ (Si–Si vibration in its crystalline phase), a low-intensity peak at 600 cm^−1^ (Si–O vibration), and a broad shoulder from 920 cm^−1^ to 980 cm^−1^ (surrounding the Si–OH vibration at 958–967 cm^−1^). All references of the Raman peaks are summarized in Table 1, with the corresponding references. The complete Raman spectra and their evolution over time, corresponding to the various experiments, are presented in the supplementary data (Figure S1).

In order to further explore the spectra, deconvolution was applied using Peakfit software. Broadening and later narrowing of the main Si–Si peaks were suspected to be due to the conversion of crystalline Si (c-Si) into amorphous Si (a-Si), followed by porous silicon degradation.

**Figure 4:**
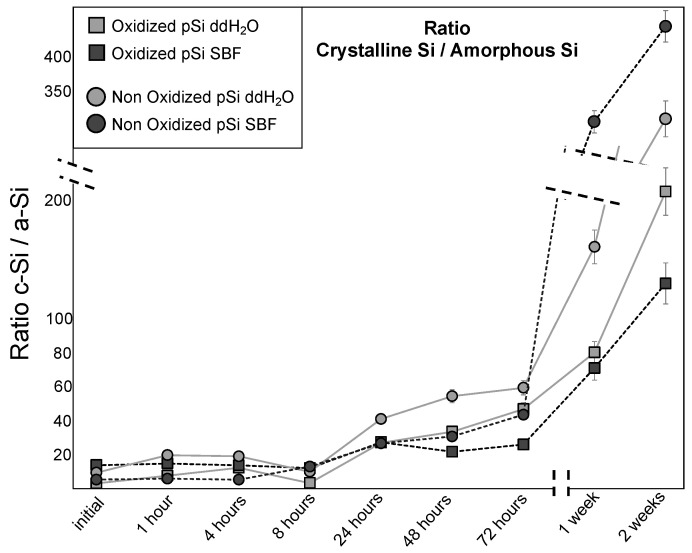
Evolution over time of the ratio cSi/aSi, from Raman peaks intensity. After 1 week, the very high ratios observed for non-Oxidized samples correspond to the disappearing of pSi layer.

In line with the literature (see Table 1), we targeted the peaks at 464, 480, and 520 cm^−1^ associated with Si–O, a-Si, c-Si, and c-Si in transverse optical mode, respectively. Thus, we could assess the ratio of c-Si peaks over an a-Si peak. This crystalline/amorphous ratio enlightens the process of resorption (when crystalline pSi transforms into its amorphous phase) and the thinning of the porous layer (when the signal of underlying crystalline bulk silicon becomes predominant). During the first day (after 1 h, 4 h and 7 h), no significant changes were noticed with a ratio ranging from 5 (Oxidized pSi after 1 h in ddH_2_O) to 15 (non-Oxidized pSi after 4 h in ddH_2_O). The ratio increased notably after 24 h, with constant increase for all samples until 72 h. After 1 week, and even more after 2 weeks, the crystalline/amorphous ratio increased dramatically, with major differences between samples (see Figure 4).

**Figure 5:**
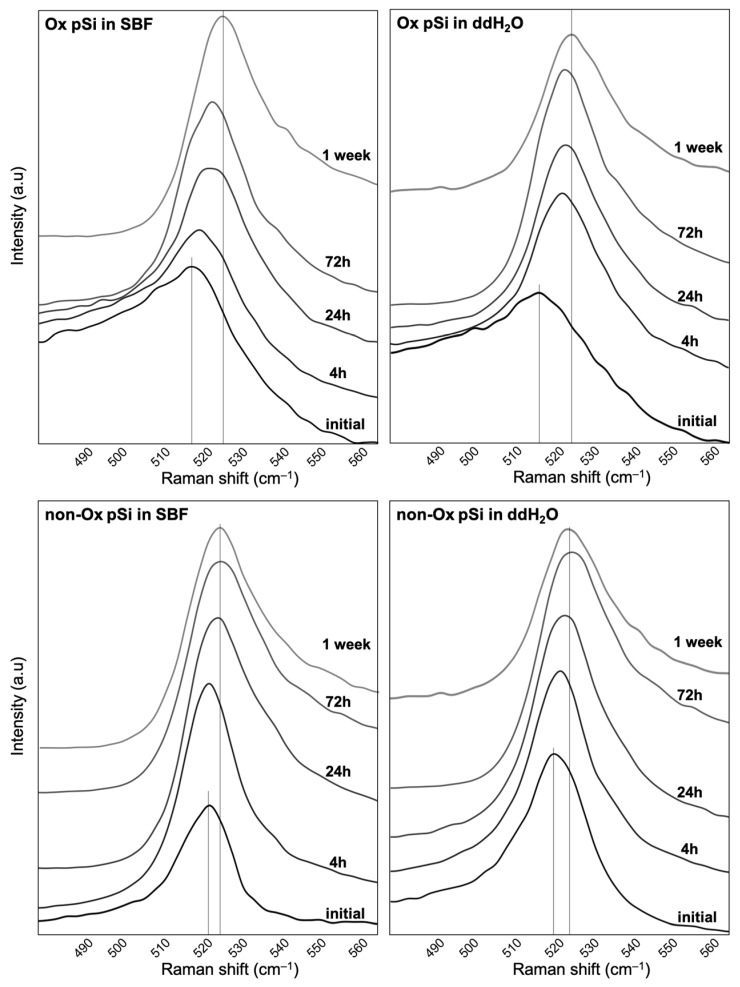
Raman shifts of the crystalline Si–Si peaks, with peak evolution over time. The main shift was obtained after 72 h for non-oxidized pSi, while it took up to 1 week for oxidized samples.

We focused on the 520 cm^−1^ peak (Si–Si vibration in its crystalline phase) to assess its shift during experiment, as its value has been described as varying according to porosity and subsequent phonon confinement). For oxidized pSi, a shift was observed towards higher wave numbers, from 515 cm^−1^ (initial samples) to 525 cm^−1^ (after 1 week in SBF or ddH_2_O). For non-oxidized pSi, a shift was observed from 520 cm^−1^ (initial samples) to 525 cm^−1^ (after only 24 h in PBS or ddH_2_O). Raman shifts of the crystalline Si–Si peaks for the various samples are presented in Figure 5.

**Figure 6:**
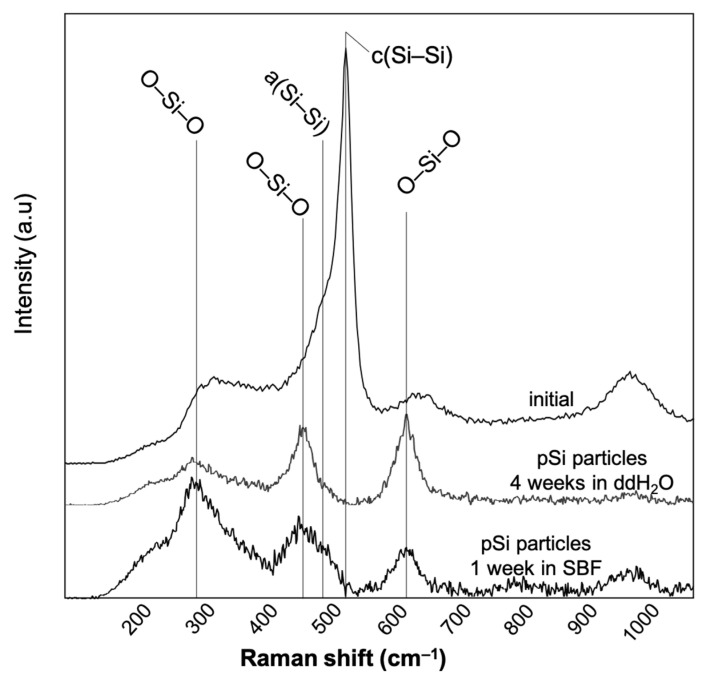
Raman spectra of non-oxidized pSi particles in ddH_2_O and in SBF. The cSi–Si peak disappeared after 1 week in SBF and after 4 weeks in ddH_2_O.

To complete the Raman spectroscopic analyses, we analyzed non-oxidized pSi particles, as they harbor obvious differences with optical microscopy. The signal/noise ratio made the spectra difficult to exploit but, after 1 week in SBF, we noticed the disappearance of the major peaks of Si–Si at 480 cm^−1^ (amorphous) and 520 cm^−1^ (crystalline), and only the peaks of Si–O (430 cm^−1^ and 465 cm^−1^) remained. Such apattern was observed for non-oxidized pSi in ddH_2_O after 4 weeks. Representative Raman spectra of non-oxidized particles are shown in Figure 6.

### 3.3. ATR-FTIR Spectroscopy

Infrared spectroscopy analysis of pSi samples during dissolution was conducted by considering the specific peaks attributed to Si–O and Si–H bonds in the range of 800 to 1200 cm^−1^. For oxidized pSi, we also assessed C=O and NH_2_ peak evolution in the range of 1300 to 1700 cm^−1^, as these samples were silanized with APTES. The major peaks considered are summarized in Table 2 with the corresponding references.

**Figure 7:**
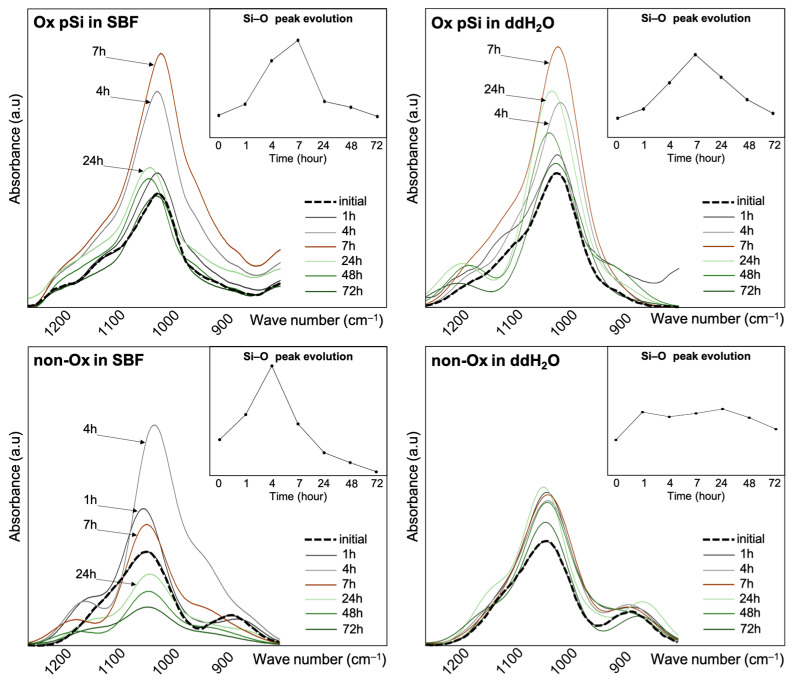
Si–O peak evolution over time, as measured via ATR-FTIR. Si–O peak increased during the first hours, before decreasing, except for non-ox pSi in ddH_2_O.

The FTIR spectra of the various samples for the region 800 to 1200 cm^−1^ are presented in Figure 7: major variations were observed on the broad absorbance peak at 1038 cm^−1^ (v(Si–O) stretching), increasing until 7 h for oxidized pSi, then slowly decreasing over time. Such variations were observed with non-oxidized samples only when placed in SBF, with an increase only until 4 h, while variations were hardly visible with non-oxidized pSi in ddH_2_O. For non-oxidized samples, we observed an initial peak at 880 cm^−1^ (δ(O–Si–H) bending), progressively disappearing after 7 h. We also noticed, only for samples placed in SBF, a shoulder corresponding to an absorbance peak at 938 cm^−1^ (δ(Si–H) deformation): this peak appeared before 7 h and was not visible anymore after 24 h.

**Figure 8:**
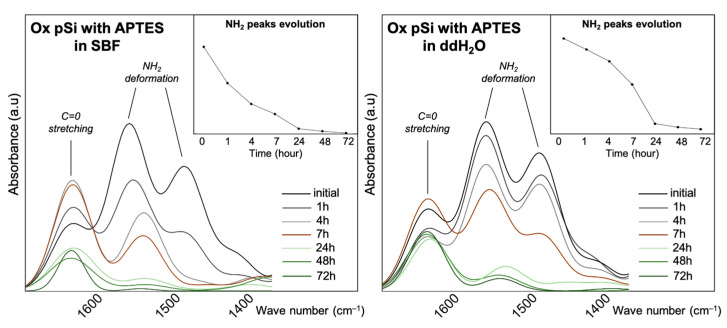
Oxidized pSi functionalized with APTES: NH_2_ peak evolution over time, as measured via ATR-FTIR. NH_2_ peak remains visible for up to 7 h in both SBF and ddH_2_O.

For oxidized pSi, we assessed APTES grafting by following absorbance peaks at 1486 cm^−1^ and 1567 cm^−1^, corresponding to NH_2_ asymmeteric and symmeteric stretching. These peaks were clearly visible at the beginning of the experiment, and disappeared progressively until 48 h where they were not visible anymore, as seen in Figure 8. As APTES was chemically grafted on the surface, the NH_2_ peaks lowering and disappearing corresponds to surface dissolution [7]. The absorbance in the area associated with *v*(Si–O) shows the oxidation of pSi and its conversion of different suboxides leading to the formation of silicic acid.

### 3.4. SEM and EDX Spectroscopy

**Figure 9:**
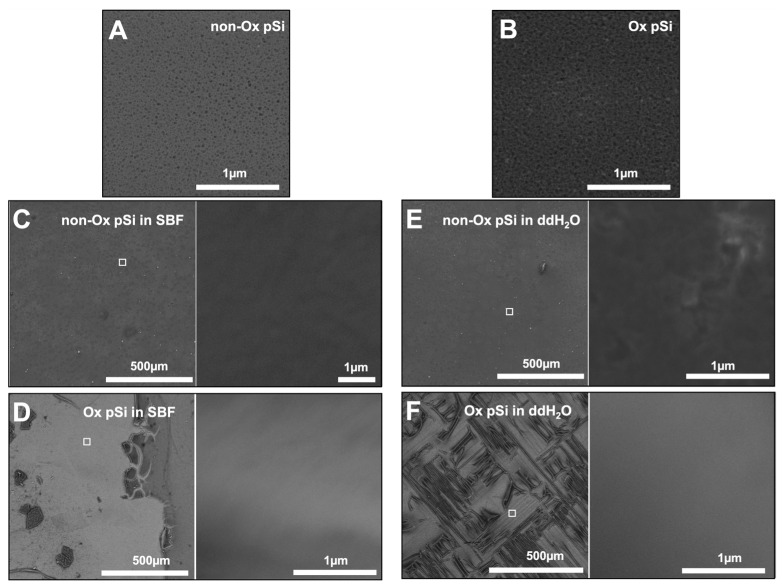
SEM of pSi chips enlightening porous layer degradation after 2 weeks. (**A**,**B**) Initial pSi layers (non-oxidized and oxidized, respectively). (**C**,**D**) pSi in SBF (non-oxidized and oxidized, respectively). (**E**,**F**) pSi in ddH_2_O (non-oxidized and oxidized, respectively). Higher magnification images in (**C**–**F**) correspond to the white square in the corresponding lower magnification images.

Scanning Electron Microscopy (SEM) measurements were used to characterize the micropore morphology changes in different samples under the study, as presented in Figure 9. The average size of the pSi micro particles is 160 ± 10 µm and the thickness is 15 ± 3 µm. The average pore diameter of the non-oxidized pSi measured via SEM is 37 ± 6 nm and that of the oxidized pSi is 30 ± 7 nm. The non-oxidized sample incubated in ddH_2_O lost the porous features all together, showing an approximately complete dissolution after 2 weeks (Figure 9E). The oxidized samples in ddH_2_O showed some remanent porous structures (Figure 9F), while in SBF, a visible porous structure could be seen with much larger pore sizes after the similar incubation time. Energy-Dispersive X-rays spectroscopy (EDX) showed an increased oxygen level in the non-oxidized samples incubated in SBF compared to the oxidized samples in SBF.

### 3.5. In Vivo Experiments

**Figure 10:**
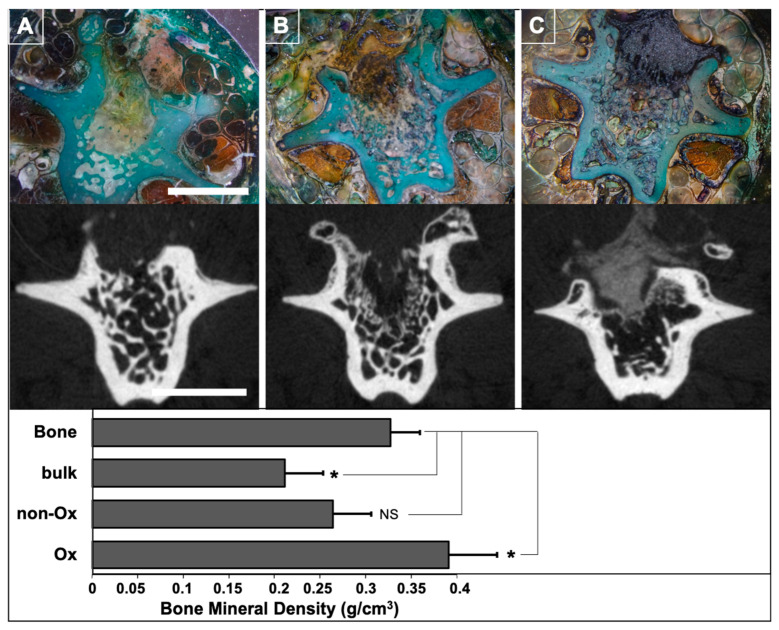
In vivo experiments conducted on rat vertebrae. Optical microscopy images of representative undecalcified sections, stained with Masson trichrome: (**A**) Oxidized pSi particles. (**B**) Non-oxidized pSi particles. (**C**) Non porous Si particles. Scale bar = 5 mm. The bottom graph represents bone mineral density, as measured from the triplicate experiments. * indicates a significant difference (*p* < 0.05). NS indicates no significant differences.

Eight weeks post-surgery, the vertebrae were analyzed via μCT and undecalcified histology to assess particle resorption and bone tissue formation. Bone mineral density (BMD), assessed via µCT, was significantly higher in vertebrae filled with oxidized pSi particles compared to non-oxidized particles (*p* < 0.01) and to non-porous particles (*p* < 0.01). Oxidized and non-oxidized pSi particles were not visible anymore on µCT, while non-porous particles were still clearly visible (Figure 10). We compared the healing bone of investigated vertebrae to spongy bone of intact vertebrae: the BMD of spongy bone was significantly higher than the BMD of defects filled with non-porous particles (*p* < 0.01), but significantly lower than the BMD of defects filled with oxidized pSi particles (*p* < 0.05). No significant difference could be observed between spongy bone and defects filled with non-oxidized pSi particles (graph in Figure 10). The histological analyses confirmed the enhanced ne bone formation for the vertebrae filled with oxidized pSi particles, with obvious particle resorption, even though there were still traces of particles undergoing dissolution, included into the regenerated bone (yellow part, in the center of the vertebrae in Figure 10A). On the contrary, the non-oxidized particles were clearly visible, in contact with newly formed bone, but not clearly surrounded by bone formation (Figure 10B). The non-porous silicon particles remained almost unaffected, and seemed to be pushed out by the bone formation (Figure 10C).

## 4. Discussion

**Figure 11:**
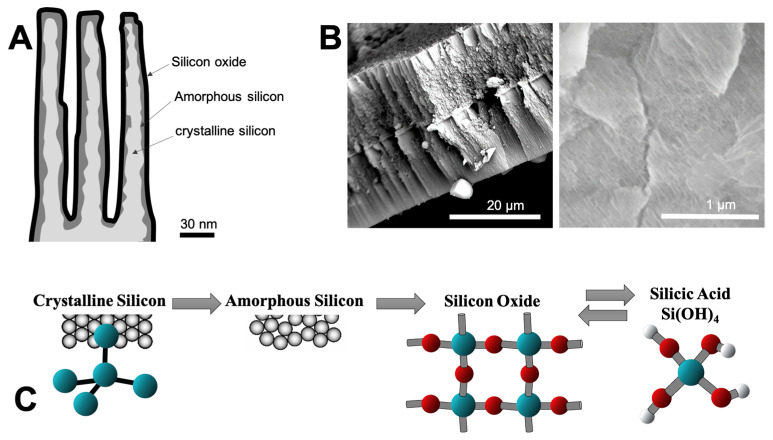
Representation of pSi dissolution, with involvement of crystalline silicon (cSi), amorphous silicon (aSi), silicon oxide (SiO_2_), and silici acid Si(OH)_4_. (**A**) Schematic representation of pSi side view, with silicon oxide and amorphous silicon layers surrounding crystalline silicon. (**B**) SEM images of pSi particles, revealing the porous part. (**C**) Psi dissolution process, from crystalline silicon to released silicic acid; blue, red, and white balls represent Silicon, Oxygen, and Hydrogen, respectively.

In the bulk silicon, there were Si–O–Si already present in the depth of the material before the etching, as evidenced by the FTIR studies. In freshly prepared pSi layers, the presence of Si–H bonds related to groups formed at the extended pSi surface were demonstrated by well-defined absorption bands at 905–910 [37]. With aging, various vibration modes were seen in the absorption spectra. This indicates that an upward frequency shift occurred during the oxidation of the porous silicon. Oxidation leads to the incorporation of oxygen into Si–Si back-bonds, leading to the formation of O_y_SiH_x_ species (schematic representation in Figure 11). The difference between the frequency of this oxidized species and the SiH_x_ is of 150–200 cm^−1^ [36].

It was demonstrated that with the progress of the synthesis of pSi, the band between 2200–2400 cm^−1^ corresponding to Si–H_x_ diminished and those attributed to Si–O–Si appeared; this is the signature of pSi oxidation [44]. The absorbance frequencies related to all the different species during the transition of pSi to porous glass are listed in Table 2.

Raman spectroscopy is a powerful yet sensitive tool that can be used to precisely detect the morphology and chemical composition of a material. Raman spectroscopy is another means to characterize pSi. Its simple process does not require special sample preparation/processing. It indicates chemical entities in a material in real-time and is especially useful to detect chemical modifications, if any. This information is reflected by the Raman spectra of the material. The changes in the Raman spectra of a nanostructured material are explained by the quantum confinement effect. Based on this effect, numerous properties of nanomaterials may be explored. The physical properties of nanomaterials like nanocrystal size, strain, temperature, conductivity, pressure effects, phase changes can be well explored by using Raman spectroscopy. 

The Raman analysis of pSi demonstrated an obvious peak for Si at 521 cm^−1^ [25,27]. Crystalline Si has a sharp peak at 520 cm^−1^, while in nano-crystalline structures, the peak shifts to below 520 cm^−1^ [28,45,46,47,48]. For the degenerated material, there is a smaller Raman shift, decreased to 505 cm^−1^, along with a broader than usual peak at 521 cm^−1^. The amorphous silicon peaked near 480 cm^−1^, this peak is usually weak in intensity and is very broad [24]. In the case of pSi, a wide range of conditions affect the peak intensity and Raman shift. The peaks may be at 510 cm^−1^, 518 cm^−1^, or 519 cm^−1^ depending upon the intrinsic structure of the material and the experimental conditions. However, it is suggested that there is resemblance between the spectra of thick film of pSi and that of the micro-crystalline Si. A band between 508–510 cm^−1^ suggests a roughly spherical structure of the porous material. Shape and polarization properties can suggest structural arrays of the silicon columns in a particular sample [24]. A narrower standard deviation of the Si peak suggests relatively small and more uniform particle size [28]. 

After electrochemical etching, if the silicon atoms in the structure maintain their local symmetry, then the similar peak as for cSi is observed. If the porous structure constitutes an array of columns, then the incident laser will be transmitted into the material and the polarized spectra would vanish in some configurations. However, for highly irregular porous structure, the transmitted light will be greatly reflected and different polarizations will give roughly same signal intensities [24].

Silicon dioxide (SiO_2_) has a non-crystalline structure containing small areas of quasi-crystalline forms. Three spectra can be recognized in SiO_2_ analysis via Raman: the main band placed between 300 cm^−1^ to 550 cm^−1^ for crystalline, D2 band around 620 cm^−1^, and another band with a maximum at 800 cm^−1^ [26]. Along with these three main bands, another group of peaks around 1000 cm^−1^ is also evident due to the sub-oxides of Si and silicic acid [30,31]. In a non-densified peak of silicon dioxide (SiO_2_), a four-member ring is at maximum at 490 cm^−1^, and at 520 cm^−1^ in a densified structure [26]. Strong lines for crystalline forms of silicon dioxide (SiO_2_) were observed from 200 cm^−1^/300 cm^−1^ to 550 cm^−1^ [26,30]. Oxides also show different oscillations, which leads to different peaks in the Raman [26]. A peak around 230 cm^−1^ or 245 cm^−1^ is referred to as scissoring of tetrahedron SiO_4/2_ [26,49,50]. The band with the peak at 320 cm^−1^ represents scissoring in extended tetrahedron of different oxidation states of silica i.e., SiO_4/2_ and SiO_4/4_. Similarly, 380 cm^−1^ shows the bending in more than five rings SiO_2_ [26]. Cristobalite, a crystalline form (polymorph) of silicon dioxide also shares a peak at 230 cm^−1^. Coesite and other polymorph quartzes have peaks at 670 cm^−1^ and around 460–465 cm^−1^, respectively [26,50].

Raman spectroscopy is a sensitive technique for the characterization of silicon and silica. The analysis of cSi and pSi is considerably clear as the band is around 520 cm^−1^ or a lower shift in the case of porous material. The analysis of SiO_2_ via Raman gives the values for Si–O, Si–OH, Si–O–Si and other sub oxides; 3-fold and 4-fold ring defects formed in the SiO_2_ network also have separate peaks. Peak frequency shift in the Raman can also estimate the stress in the films of the material [46]. Different levels of oxidation along with variations in pore size, particle size, and uniformity of the two can result in shifts in the Raman peaks. As the density of the material decreases, the characteristic peak shifts to the lower value. Three main changes occurring in the Raman spectra are broadening, shifting to a lower wavelength, and asymmetry. The shift of the classical first order Raman spectrum of bulk/flat silicon at 521 cm^−1^, that originally immerge due to the excitation of optical phonons close to Γ point of Brillouin zone O(Γ), to the lower wavenumbers can be explained by the phonon confinement model specific to the crystalline lattice of silicon [51]. The presence of the small crystalline lattice in pSi as compared to the flat-Si is responsible for this shift. In bulk silicon, the Raman scattering on phonons occurs when the photon with a low momentum probes the optical phonon close to central Γ point (k = 0) of Brillouin zone O(Γ). In this scattering the momentum (q) and wavevector (k) are conserved [52]. Thus, the first-order Raman spectra due to transverse optical mode at 521 cm^−1^ is of a Lorentzian shape [53].

In the case of nano-crystallites of porous silicon, the geometric extensions/dimensions of the crystal decreases, which lifts this rule for low-momentum photon excitation; owing to phonon localization, and scattering occurs due to probing of more phonon with larger momentum from a broader interval around Γ. The localization of optical phonon in a finite crystal is determined by a weighting function over k in the first Brillouin zone [54]. The amplitude of the phonon is reduced as it moves from the crystal center to its boundary. Hence, the strongest contribution is from the frequencies near the Γ-point [51]. Raman scattering intensity is the superposition of Lorentzian curves centered at different wavenumbers [55]. With different weights, the Raman peak is shifted towards lower wavenumbers and the spectrum shape becomes broader and asymmetric with decrease in nanocrystal size [53,54]. Furthermore, this shift is also associated with the broadening that is due to the width of the peak, indicating the degradation of the nanocrystals and the appearance of a-Si phase [56]. In the case of amorphous silicon, the long-range transitional symmetry of the crystal is lost and all the associated principles are no longer followed and the spectrum of an amorphous silicon is actually the sampling all the modes in the Brillouin zone.

Oxidation of pSi, thermally decreases the size of the pore to due to microcrystalline changes in the structure and the surface termination by the hydrogen atom is replaced by a hydroxyl group. After incubation in PBS, the increase in the oxygen content of the non-oxidized sample shows that there is more degradation of this sample as compared to the oxidized samples. SEM pictures showing increased pore size in case of samples incubated in PBS show the interlacing of the pore during resorption. More swift dissolution and loss of porous of non-oxidized samples in water are concurrent with the findings of increased release of silicic acid from the same sample in water via ICP-MS spectroscopy.

Taken altogether, the in vitro experiments highlight that non-oxidized pSi underwent high chemical modification, especially in SBF, with an accelerated initial dissolution in ddH_2_O and an inhibition of initial Si release in SBF (Figure 3). This high reactivity also led to the transformation towards amorphous non-resorbable silica when incubated in SBF (Figure 2). In comparison, the resorption process of oxidized pSi seemed delayed but more continuous (Figure 2, Figure 3 and Figure 4). The surface chemical modification with APTES grafting was maintained for several hours (Figure 7). And, as it has previously been shown that APTES grafting enhances cell adhesion and biological integration [7], the persistency of APTES for some hours renders pSi use interesting for in vivo implantation, in order to have an effect during the initial integration after grafting.

These observations were confirmed in vivo with the animal experiments: oxidized pSi were almost completely resorbed after 2 months of healing, with remaining partially dissolved particles surrounded by newly formed bone. Surprisingly, non-oxidized particles were still obviously present after 2 months with limited bone regeneration. This delayed resorption is consistent with the in vitro observations in SBF, and particles transformation towards silica. Non-porous Si particles were used as non-resorbable control, and did not allow satisfying bone regeneration: they seemed to be pushed out from the defect, enlightening the importance of Si particles resorption during the bone healing process [57]. The µCT analyses were consistent with the pSi resorption processes: the continuous predictable resorption of oxidized pSi particles led to a significantly increased BMD, even higher than the original trabecular bone.

## 5. Conclusions

Our findings demonstrate the timeline for the complete degradation of pSi, along with evidence of silicic acid release. Interestingly, the time frame of complete in vitro degradation of oxidized pSi was reproduced during in vivo experiments. Via FTIR and Raman spectroscopy, we were able to follow the process of resorption through the intensity of Si–O peaks and crystalline and amorphous Si peaks. In parallel, the degradation studies, evaluated by ICP-MS, revealed a constant release of silicic acid for several weeks, with maximum release during the first 48 h. The in vitro degradation of non-oxidized pSi was much more accelerated than that of oxidized pSi, but its high reactivity in SBF led to transformation in silica and prevented its degradation in vivo, thus limiting its biointegration and further bone formation. As it is well known that silicic acid is a bioactive compound; its release by pSi particles enhanced bone regeneration. A constant supply of silicic acid is required over the period of bone regeneration, and, as oxidized pSi degrades slowly, it provided a constant surge of silicic acid over the time and increased the amount and density of regenerated bone.

## Figures and Tables

**Table 1:** Raman peaks: Raman shift and its corresponding molecular bonding, according to data available in the literature.

Wavenumber (cm^−1^)	Raman Assignment	References
480	Amorphous silicon	[24]
508–510	Porous silicon	[24]
518	Light emitting porous silicon	[24]
520/521	Crystalline Silicon	[24,25,26,27,28]
535–540	Crystalline silicon (TO)	[29]
600/620 (D2)	3-member ring in SiO_2_ structure (Densified bulk SiO_2_)	[30]
958/967	Si–OH	[30,31]
1097	Si–O	[31]

**Table 2:** FTIR bands (absorbance) and corresponding atomic liaisons, according to data available in the literature.

Absorbance (cm^−1^)	Species	Vibrations	References
640	–SiH_x_ (x = 3,2,1)	δ(Si–H) wagging	[32]
880	OySiH_x_	δ(O–Si–H) bending	[33]
905	Si–H	δ(Si–H) deformation	[33]
938	Si–H	δ(Si–H) deformation	[27]
1038	Si–O	v(Si–O) stretching	[34]
1056	Si–O	v(Si–O) stretching	[35]
1065	Si–O–Si	v(Si–O) stretching	[36,37]
1105, 1150, 1100	Si–O–Si	v(Si–O–Si) asymmetric stretching	[35,36,38,39]
1300	Si–O–Si	Deformation mode	[40]
1411	Si–CH_2_	Deformation mode	[41]
1486, 1567, 1600	NH_2_	Asymmeteric and symmeteric stretching	[41,42]
2080	Si_3_Si–H	Stretching vibration	[43]
2187	O_3_Si–H	Stretching vibrations	[43]

## Data Availability

The data presented in this study are available on request from the corresponding author. The raw/processed data required to reproduce these findings cannot be shared at this time due to technical limitations.

## Supplementary Materials

The following supporting information can be downloaded at: https://www.mdpi.com/article/10.3390/jfb14100493/s1, Figure S1: Raman spectra and their evolution over time.
