# Supplementary figures and images for: Nanostructured Porous Silicon for Bone Tissue Engineering: Kinetics of Particle Degradation and Si-Controlled Release
Source: J Funct Biomater. 2023 Sep 30;14(10):493. doi: 10.3390/jfb14100493 (PMC10607156; doi:10.3390/jfb14100493)

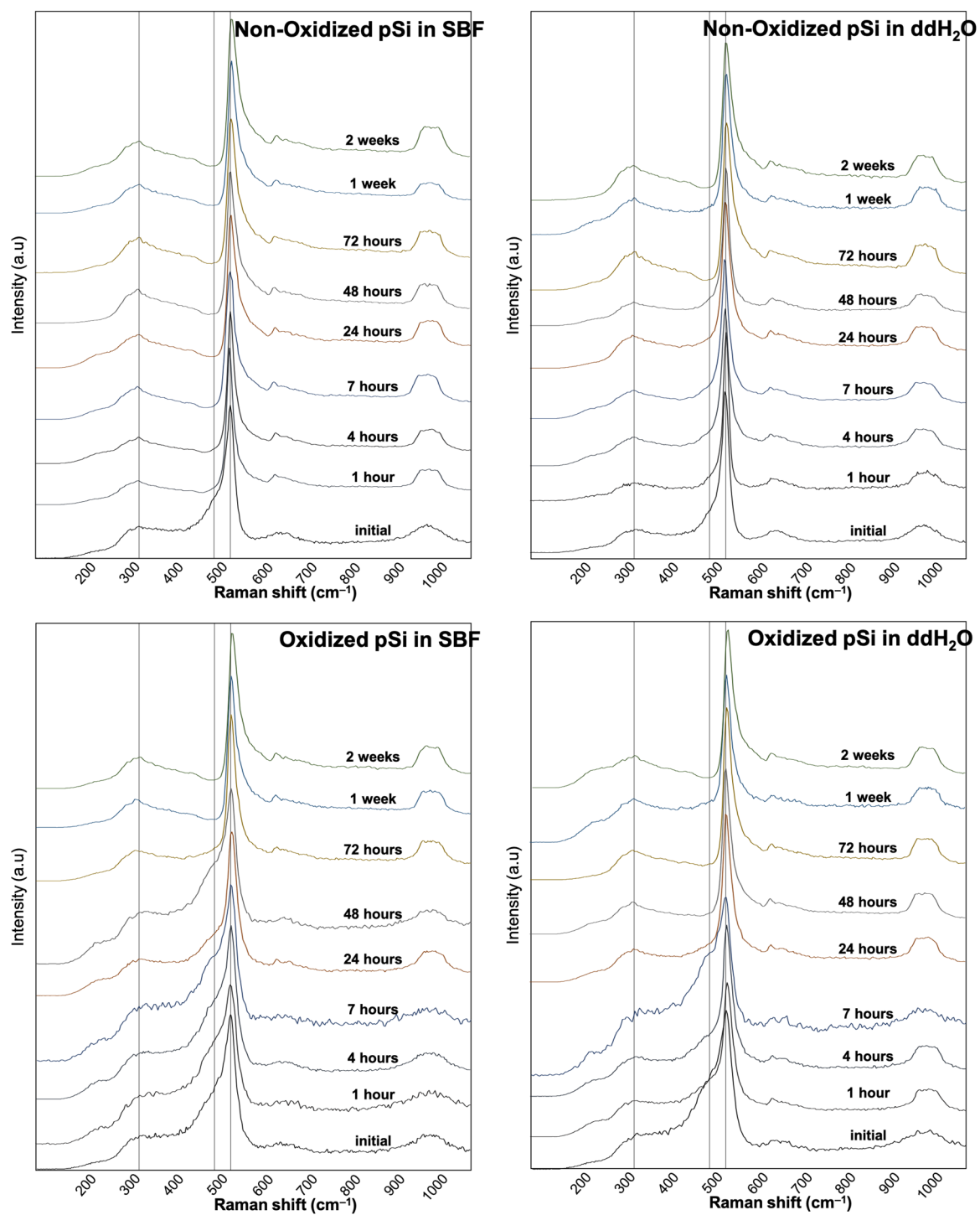

**Figure S1:** Raman spectra and their evolution over time.

Supplement: Supplementary file 1 [file jfb-14-00493-s001.zip › jfb-2571912-supplementary.pdf]
